# Spinal Manifestation of Malignant Primary (PLB) and Secondary Bone Lymphoma (SLB)

**DOI:** 10.3390/curroncol28050332

**Published:** 2021-10-02

**Authors:** Melanie Barz, Kaywan Aftahy, Insa Janssen, Yu-Mi Ryang, Georg Prokop, Stephanie E. Combs, Philipp J. Jost, Bernhard Meyer, Jens Gempt

**Affiliations:** 1 Department of Neurosurgery, Klinikum rechts der Isar, School of Medicine, Technical University Munich, 81675 Munich, Germany; kaywan.aftahy@tum.de (K.A.); insajanssen@icloud.com (I.J.); Yu-Mi.Ryang@helios-gesundheit.de (Y.-M.R.); bernhard.meyer@tum.de (B.M.); jens.gempt@tum.de (J.G.); 2 Department of Neurosurgery, Hôpitaux Universitaires de Genève, 1205 Geneva, Switzerland; 3 Department of Neurosurgery, Helios Klinikum Berlin-Buch, 13125 Berlin, Germany; 4 Department of Neuropathology, Institute of Pathology, Klinikum rechts der Isar, School of Medicine, Technical University Munich, 81675 Munich, Germany; georg.prokop@tum.de; 5 Department of Radiation Oncology, Klinikum rechts der Isar, School of Medicine, Technical University Munich, 81675 Munich, Germany; stephanie.combs@tum.de; 6 Helmholtz Zentrum Munich, Department of Radiation Sciences (DRS), Institute of Radiation Medicine (IRM), 85764 Munich, Germany; 7 German Cancer Consortium (DKTK), Partner Site Munich, 80336 Munich, Germany; 8 Medical Department III for Hematology and Oncology, Klinikum rechts der Isar, School of Medicine, Technical University Munich, 81675 Munich, Germany; philipp.jost@tum.de; 9 Division of Oncology, Department of Internal Medicine, Medical University of Graz, 8036 Graz, Austria

**Keywords:** malignant lymphoma, bone lymphoma, spine surgery, diffuse large B-cell lymphoma, epidural compression

## Abstract

Manifestation of malignant lymphoma in the spine is rare; there have only been a few cases reported in the literature. Due to its rarity, there is no gold standard for the management of patients suffering from spinal lymphoma manifestations. Methods: We retrospectively reviewed the data for 37 patients (14 female, 23 male) with malignant lymphoma in the spine receiving intervention in our center from March 2006 until June 2020. Neurological impairment, pain, diagnostics, and/or surgical instability were the criteria for surgery in this patient cohort. Otherwise, only CT-guided biopsies were conducted. Analysis of the patient cohort was based on the Karnofsky performance status scale (KPSS), location of the lesion, spinal levels involved, spinal instability neoplastic score (SINS), surgical treatment, histopathological workup, adjuvant therapy, and overall survival. The following surgical procedures were performed: posterior stabilization and decompression in nine patients; decompression and/or tumor debulking in 18 patients; a two-staged procedure with dorsal stabilization and vertebral body replacement in four patients; decompression and biopsy in one patient; a two-stage procedure with kyphoplasty and posterior stabilization for one patient; posterior stabilization without decompression for one patient; a vertebroplasty and cement-augmented posterior stabilization for one patient; and a CT-guided biopsy alone for two patients. Twenty-one patients (56.78%) had ≥1 lesion in the thoracic spine, 10 patients (27.03%) had lesions in the lumbar spine, two patients had lesions in the cervicothoracic junction, two patients had lesions in the thoracolumbar junction, one patient had a lesion in the lumbosacral junction, and one patient had a lesion in the sacrum. The diagnoses of the histopathological workup were diffuse large B-cell lymphoma in 23 (62.16%) cases, indolent lymphoma in 11 (29.74%) cases, anaplastic T-cell lymphoma in one case (2.70%), T-cell lymphoma in one case (2.70%), and Burkitt lymphoma in one (2.70%) case. The median overall survival was 7.2 months (range 0.1–266.7 months). Pre- and postoperative KPSS scores were 70% (IQR 60–80%). Manifestation of malignant lymphomas in the spine is rare. Similar to the approach taken for spine metastases, a surgical intervention in cases of neurological impairment or manifest or potential instability is indicated, followed by chemoimmunotherapy and radiotherapy.

## 1. Introduction

Primary bone lymphoma (PLB) is a rare disease, occurring in only 2% of all bone tumors and only 5% of all extra-nodal lymphomas [1,2]. To mitigate inconsistencies in the definition of a PLB, Ostrowski et al. developed guidelines stating that lymphomas can be considered PLBs when the disease affects one or more bones with or without involvement of local lymph nodes but with no evidence of disease in distant nodes or other extraosseous sites, as specified in the Ann Arbor classification stages I and II [3]. According to the WHO’s 2017 classification of bone and soft tissue tumors, secondary bone lymphomas (SLBs) are defined as bone lesions with evidence of systemic disease or a primary soft tissue mass secondarily involving a bone [4].

Alencar et al. showed that the majority of bone lymphomas are non-Hodgkin’s lymphoma (NHL); in particular, the subtype diffuse large B-cell lymphoma (DLBCL) appears to be over-represented [5]. Long bones such as the femur are the most commonly involved anatomic sites, followed by the vertebrae column and pelvis [1,6,7,8].

Similar to patients with other types of tumor histology, patients with bone lymphoma present with debilitating pain and/or neurological impairment resulting from spinal cord compression or loss of spinal integrity [9,10,11,12,13,14]. There are many treatment recommendations for this type of disease, such as chemotherapy, immunotherapy, surgery, or radiotherapy. After the radiosensitivity of lymphoma was demonstrated by a few research groups, current evidence still supports radiotherapy, even in cases of progressive neurological impairment, and the role of surgical intervention is still a controversial debate [10,11,12,13,14]. There have been a few case studies, such as those from Laufer and Love, presenting neurologic recovery in cases of urgent surgical decompression followed by radiotherapy [13,14]. Due to its rarity, there is no gold standard to date for the management of patients suffering from spinal lymphoma manifestations.

## 2. Materials and Methods

### 2.1. Patients and Methods

Data from 37 patients who underwent intervention for PLB or SLB in the spine between March 2006 and June 2020 were analyzed retrospectively and met the following inclusion criteria: surgery for PLB or SLB in the spine, neurological impairment, pain, need of diagnostics, and/or spinal instability. Furthermore, preoperative age, sex, Karnofsky performance status scale (KPSS) score, location of the lesion, surgical treatment, spinal instability neoplastic score (SINS), histopathological workup, adjuvant therapies, and overall survival were recorded for each patient.

Surgery was performed by neurosurgeons experienced in the treatment of spinal tumors. The decisive factors were neurological impairment as measured by the American Spinal Injury Association (ASIA) Impairment Scale (AIS) and spinal instability.

### 2.2. Statistics

Statistical analysis was performed using STATA Version 13.1 (2011, StataCorp, College Station, TX, USA). In the descriptive data analysis, we show non-normally distributed data as median and interquartile range (IQR), and normally distributed variables as mean and standard deviation. 

### 2.3. Ethics Approval

The study was approved by the local ethics committee (335/16S) of the Technical University Munich, School of Medicine. It was conducted in accordance with the ethical standards of the 1964 Declaration of Helsinki and its later amendments [15].

## 3. Results

Thirty-seven patients (23 male, 14 female) with a median age of 71.0 years (range 24–84 years) at surgery met our inclusion criteria. The median overall survival was 7.2 months (range 0.1–266.7 months). Preoperative KPSS was 70% (IQR 60–80%), as was the postoperative KPSS. Twenty-seven patients presented with PLB, and 10 patients presented with SLB (Table 1).

Besides the clinical data, the imaging data were evaluated as well; 21 patients (56.78%) had ≥1 lesion in the thoracic spine, 10 patients (27.03%) had lesions in the lumbar spine, two patients had lesions in the cervicothoracic junction, two patients had lesions in the thoracolumbar junction, one patient had a lesion in the lumbosacral junction, and one patient had a lesion in the sacrum (Table 2).

**Figure 1:**
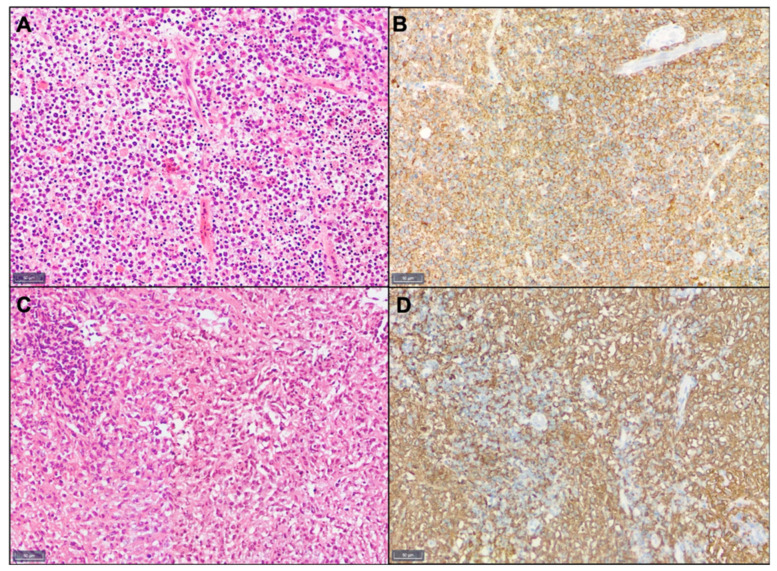
Example of histopathology. Shown are HE (**A**,**C**) and CD20 immunohistochemistry (**B**,**D**) of a manifestation of a diffuse large cell lymphoma (**A**,**B**) and a follicular lymphoma (**C**,**D**). Scale bars: 50 μm.

Indications for surgery were neurological impairment in nine cases, pain in 19 cases, need of diagnostic tissue samples in two cases, and spinal instability in 21 cases (Figure 1). Based on these cases, 16 patients showed a combination of pain and neurological deficit. Seven patients showed a combination of pain, neurological deficit, and spinal instability. The SINS was used to verify the indication for surgery in cases of spinal instability. The cohort presented with a median SINS of 7. Thirteen patients were considered to have stable spines, and 24 had indeterminate instability (SINS 7–12). Nine patients received posterior stabilization and decompression, 18 received decompression and/or tumor debulking, four underwent a two-stage procedure with dorsal stabilization and vertebral body replacement, one received decompression and a biopsy, one received a two-stage procedure of kyphoplasty and posterior stabilization, one underwent posterior stabilization without decompression, one received vertebroplasty and cement-augmented posterior stabilization, and two patients received a CT-guided biopsy only.

Postoperatively, complications occurred in seven patients (Table 3). One patient died during the inpatient stay as a result of a pulmonary artery embolism.

In 34 patients, surgical treatment was followed by systemic chemoimmunotherapy and/or radiotherapy (Table 4). Two patients decided on palliative care without any adjuvant treatment, and seven patients died during follow-up. The histopathological work-up resulted in DLBCL (62.16%) as the most common subtype, followed by follicular lymphoma (18.92%) (Table 5) (Figure 1). Clinical information at 6-month follow-ups were available in 16 patients. Eight patients died during follow-up.

## 4. Discussion

Malignant non-Hodgkin lymphoma (NHL), accounting for nearly 5% of all adult cancers, represent a group of heterogeneous neoplasias arising in the lymph nodes or bone marrow. Two classification categories for NHL exist: PLBs and disseminated lymphomas with infiltration of the bone [16].

Although disseminated lymphoma with secondary involvement of the axial skeleton is not uncommon, PLB is very rare, comprising only 7% of primary malignant bone tumors and only 5% of extranodal lymphomas [17,18,19]. An analysis of 37 patients showed that 27 patients presented with PLB. This could be because we only treat patients with lymphoma for neurological deficits and/or spinal pain. All other patients with this disease remain in the Hematooncology Department. In all 37 cases, the most common subtype of NHL was DLBCL in the thoracic spine, followed by follicular lymphoma, as previously published by different research groups [14,20,21]. At the current time, this is the largest study cohort to date with these different types of lymphoma.

Literature still refers to conservative treatment for malignant spinal lymphoma due to the responsiveness of lymphomatous spinal lesions to systemic therapy [10,11,12,14,20,22,23]. Even in the case of neurological impairment without spinal instability, radiotherapy is still the first line of treatment [11,12,20]. There are only a few case reports exploring the outcome of surgical intervention. In this retrospective study, we have presented the demographic factors, surgical indication, treatment, and outcomes of 37 patients divided into groups—10 patients with SLB and 27 patients with PLB. To date, there is no standardized treatment guideline except in the case of spinal instability [13]. In these cases, identified by the SINS, there is a need for surgical intervention to prevent further rapid neurological impairment and to retain spinal stability [13]. Twenty-one of our patient cohort presented with indeterminate SINS and received posterior stabilization. Of this cohort, three patients improved by at least one grade according to ASIA classification; the others maintained their neurological status.

In cases of stable SINS with neurological deficits and/or pain due to tumor mass with spinal cord compression, we performed tumor debulking and/or decompression by laminectomy.

Our cohort recovered from their neurological deficiencies or reported their pain remaining the same, as described by the research group of Patchell et al. [24].

In contrast, Székely et al. published a series of 13 cases of epidural lymphoma and found limited improvement in seven patients, whereupon the authors emphasized the importance of interdisciplinary consultation and weighing individual priorities in indications for surgery, but they also emphasized urgent surgical decompression in cases of serious neurological disorders, as they were often the first clinical signs of spinal presentation of malignant lymphomas [25]. On the other hand, Peng et al. reported that six of eight patients fully recovered after surgical decompression regarding their neurological impairment [26]. Others who have reviewed their patient data have come to similar conclusions [9,11,27,28,29,30,31].

Taking these factors into consideration, rapid neurologic deterioration (independent of etiology) is advocated as indication for operative decompression. Additionally, surgery enables open biopsy for diagnosis and further adjuvant therapies, as described in 1996 by Schwechheimer et al. [32].

Various other research groups have shown that surgical treatment without adjuvant therapy is not beneficial for overall or progression-free survival [33,34,35].

In our cohort, only two patients had no adjuvant therapy, and after six months of follow-up, only four patients showed new lesions of the lymphoma; two of them were spinal.

Due to the lack of comparability of lymphomas outside the spine in our cohort, it is not possible for us to make a statement regarding prognosis. However, Wang et al. showed in 2019 that lymphomas in the spine have a significantly worse prognosis than lymphomas in the rest of the skeleton [8].

In summary, a multimodal concept should be implemented in regard to surgical treatment and subsequent adjuvant therapy.

## 5. Conclusions

Manifestation of malignant lymphomas in the spine is rare. Similar to the approach taken for spine metastases, a surgical intervention in cases of neurological impairment or manifest or potential instability is indicated, followed by chemotherapy and radiotherapy. Surgical treatment is safe and improves on the preoperative functional neurological status. Interdisciplinary consultation and decisions are unconditionally recommended.

## Figures and Tables

**Table 1:** Demographic and clinical data overview. KPSS: Karnofsky performance status scale; ASIA: American Spinal Injury Association; SINS: spinal instability neoplastic score. m: male; f: female.

Demographics *N* (%) or Mean/Median	Primary Spinal Lymphoma	Secondary Bone Lymphoma	Total
Age	71.0 years	59.1 years	71.0 years
Sex	15 m/12 f	8 m/2 f	23 m/14 f
KPSS	70%	80%	70%
ASIA A	1 (3.70%)	2 (20.00%)	3 (8.11%)
ASIA B	3 (11.11%)	0	3 (8.11%)
ASIA C	3 (11.11%)	0	3 (8.11%)
ASIA D	15 (55.56%)	4 (40.00%)	19 (51.35%)
ASIA E	5 (18.52%)	4 (40.00%)	9 (24.32%)
SINS, *N* (%)			
Median	7.0	7.0	7.0
Mean	7.1	7.2	7.1
Stable	10 (37.03%)	3 (30.00%)	13 (35.13%)
Indeterminate	17 (62.97%)	7 (70.00%)	24 (64.87%)
Clinical presentation postoperative			
KPSS	70%	80%	70%
ASIA A	1 (3.70%)	2 (20.00%)	3 (8.11%)
ASIA B	2 (7.41%)	0	2 (5.41%)
ASIA C	1 (3.70%)	0	1 (2.70%)
ASIA D	14 (51.85%)	4 (40.00%)	18 (48.65%)
ASIA E	9 (33.33%)	4 (40.00%)	13 (35.14%)
Overall survival in months	7.3	5.4	7.2

**Table 2:** Localization of tumor lesions.

Location *N* (%) or Mean/Median	Primary Spinal Lymphoma	Secondary Bone Lymphoma	Total
Thoracic	16 (59.26%)	5 (50.00%)	21 (56.76%)
Lumbar	8 (29.63%)	2 (20.00%)	10 (27.03%)
Sacral	0	1 (10.00%)	1 (2.70%)
Cervico-thoracic	1 (3.70%)	1 (10.00%)	2 (5.41%)
Thoraco-lumbar	1 (3.70%)	1 (10.00%)	2 (5.41%)
Lumbo-sacral	1 (3.70%)	0	1 (2.70%)

**Table 3:** Presentation of postoperative complications.

Postoperative Complications *N* (%) or Mean/Median	Primary Spinal Lymphoma	Secondary Bone Lymphoma	Total
Pulmonary embolus	1 (3.70%)	0	1 (2.70%)
Deep vein thrombosis	3 (11.11%)	0	3 (8.11%)
Wound infection	1 (3.70%)	1 (10.00%)	2 (5.41%)
Epidural hematoma	1 (3.70%)	0	1 (2.70%)

**Table 4:** Adjuvant therapy divided according to the primary spinal lymphoma and secondary bone lymphoma.

Adjuvant Treatment *N* (%) or Mean/Median	Primary Spinal Lymphoma	Secondary Bone Lymphoma	Total
Chemoimmunotherapy alone	12 (44.44%)	7 (70.00%)	19 (51.35%)
Radiotherapy alone	3 (11.11%)	0	3 (8.11%)
Chemoimmunotherapy + radiotherapy	5 (18.52%)	1 (10.00%)	6 (16.22%)
Unknown	5 (18.52%)	1 (10.00%)	3 (8.11%)
No treatment	2 (7.41%)	1 (10.00%)	3 (8.11%)

**Table 5:** Histopathological diagnosis divided according to frequency. DLBCL: diffuse large B-cell lymphoma.

Histopathological Workup *N* (%) or Mean/Median	Primary Spinal Lymphoma	Secondary Bone Lymphoma	Total
DLBCL	18 (66.67%)	5 (50.00%)	23 (62.16%)
Follicular lymphoma	4 (14.81%)	3 (30.00%)	7 (18.92%)
Marginal zone lymphoma	1 (3.70%)	1 (10.00%)	2 (5.41%)
Burkitt lymphoma	1 (3.70%)	0	1 (2.70%)
Mantle cell lymphoma	2 (7.41%)	0	2 (5.41%)
Anaplastic T-cell lymphoma	1 (3.70%)	0	1 (2.70%)
T-cell DLBCL	0	1 (10.00%)	1 (2.70%)

## Data Availability

Data and material are not publicly available. The data for this manuscript can be obtained from the author upon reasonable request.
